# *Haemophilus Influenzae* Microarrays: Virulence and Vaccines

**DOI:** 10.1002/cfg.194

**Published:** 2002-08

**Authors:** Tahir R. Ali, J. Simon Kroll, Paul R. Langford

**Affiliations:** Department of Paediatrics Faculty of Medicine Imperial College of Science, Technology and Medicine St Mary's Campus London W2 1PG UK

## Abstract

In 1995 the genome sequence of the *Haemophilus influenzae* KW20 (Rd) strain was published, the first available for a free-living organism. The genome has been invaluable in global strategies to identify certain virulence-related genes, e.g. those involved in LPS synthesis, and also essential genes, but there is a paucity of wholegenome transcriptome studies. We have now constructed a whole-genome array consisting of genes from Rd, additional genes identified in other strains of *H. influenzae* and controls (from eukaryotic sources and other bacteria). We intend to use this array in studies aimed at understanding the bacterium’s basic metabolism and its response to changing environments; deciphering global regulatory networks (by comparison of wild-type and mutant strains); and identifying genes expressed *in vivo*. The use of *H. influenzae* DNA arrays combined with proteomic approaches will enhance our understanding of the metabolism and virulence of the organism. Additionally, the genome sequence of a non-typable *H. influenzae* strain is in progress. The sequence from this isolate will be invaluable not only in identifying potential novel antibiotic targets and putative vaccine candidates but also in the design of a microarray for genome-typing purposes.


**Comparative and Functional Genomics**
*Comp Funct Genom* 2002; **3**: 358–361.
Published online in Wiley InterScience (www.interscience.wiley.com). **DOI:** 10.1002/cfg.194

Conference Review

### *Haemophilus influenzae* microarrays: virulence and vaccines

Tahir R. Ali, J. Simon Kroll and Paul R. Langford*
*Department of Paediatrics, Faculty of Medicine, Imperial College, St Mary's Campus, London W2 1PG, UK*

*Correspondence to:
Paul R. Langford, Department of Paediatrics, Faculty of Medicine, Imperial College of Science, Technology and Medicine, St Mary's Campus, London W2 1PG, UK.
E-mail: p.langford@ic.ac.uk*

Received: 31 May 2002
Accepted: 12 June 2002

### Abstract

**In 1995 the genome sequence of the *Haemophilus influenzae* KW20 (Rd) strain was published, the first available for a free-living organism. The genome has been invaluable in global strategies to identify certain virulence-related genes, e.g. those involved in LPS synthesis, and also essential genes, but there is a paucity of whole-genome transcriptome studies. We have now constructed a whole-genome array consisting of genes from Rd, additional genes identified in other strains of *H. influenzae* and controls (from eukaryotic sources and other bacteria). We intend to use this array in studies aimed at understanding the bacterium's basic metabolism and its response to changing environments; deciphering global regulatory networks (by comparison of wild-type and mutant strains); and identifying genes expressed *in vivo*. The use of *H. influenzae* DNA arrays combined with proteomic approaches will enhance our understanding of the metabolism and virulence of the organism. Additionally, the genome sequence of a non-typable *H. influenzae* strain is in progress. The sequence from this isolate will be invaluable not only in identifying potential novel antibiotic targets and putative vaccine candidates but also in the design of a microarray for genome-typing purposes. Copyright © 2002 John Wiley & Sons, Ltd.**

**Keywords:** ***Haemophilus influenzae*; microarray; virulence; vaccine; transcriptome; genome**

*Haemophilus influenzae* is a non-motile Gram-negative bacterium with fastidious growth requirements. Capsulate strains, and in particular type b strains with a polyribosyl ribitol phosphate capsule, can cause life-threatening infections, including meningitis, sepsis and epiglottitis. However, the availability for the past 15 years of a highly effective vaccine has led to a dramatic decrease in disease caused by type b strains in the developed world, although infections are still a significant problem in the developing world. Currently, considerable research effort is being devoted to non-typable *H. influenzae* strains (strains that do not possess a capsule), since they are a major cause of respiratory tract infection, including otitis media, sinusitis and pneumonia, as well as exacerbation of disorders such as chronic bronchitis or cystic fibrosis. Research is being driven by the need to understand pathogenesis in order to produce a vaccine effective against diverse strains.

A significant claim to fame is that a strain of *H. influenzae* (KW 20, Rd) was the first free-living organism to have its whole genome sequenced [4]. The 1,830,138 bp genome (Genbank Accession No. L42023) is predicted to encode 1714 proteins. Among many features of interest are the comparative paucity of two-component systems (four sensors/five regulators compared to 40 sensor–regulator pairs in *Escherichia coli*); the lack of three enzymes of the tricarboxylic acid (TCA) cycle (citrate synthase, isocitrate dehydrogenase and aconitase); and, as has been found subsequently in many prokaryotic genomes, approximately one-third of genes — unique or conserved hypotheticals — being of unknown function. A comprehensive comparison of the *H. influenzae* genome with that of *E. coli* was made by Tatusov

Copyright © 2002 John Wiley & Sons, Ltd.

*et al.* [9]. These authors highlighted striking differences in both the repertoire of genes and their arrangement in the two bacterial chromosomes. In particular the 2.5-fold reduction in the number of genes in *H. influenzae* compared with *E. coli* is partly a result of the absence of many functional systems, notably pathways of carbohydrate utilization and respiratory chains. Their conclusion was that the repertoire of metabolic enzymes in *H. influenzae* was tuned towards reducing conditions — that the bacterium has a largely anaerobic metabolism. It should be noted that Rd lacks approximately 300 kb of chromosomal DNA found in clinical isolates. Some of these 'missing' genes are known, e.g. fimbrial and capsulation loci [4], and remarkably, capsulation genes alone can convert non-virulent Rd to being as virulent as wild-type strains in the infant rat model [10].

To date, comparatively few studies have made extensive use of the genomic information obtained from Rd. Notable exceptions are the studies of Moxon and colleagues [6,7] and Akerley *et al.* [1]. In the former studies the Rd genome was interrogated and, by comparison with sequences of known LPS biosynthetic genes from other organisms, 25 candidate LPS genes were identified and cloned. Virulence studies in the infant rat of wild-type and knock-out mutant strains allowed the minimal LPS structure required for intravascular dissemination to be determined [6]. In the second study [7], the Rd genome sequence was searched to identify nine novel loci with multiple (range 6–36, mean 22) tandem tetranucleotide repeats. All were found to be located within putative open reading frames (ORFs), which included a homologue of the neisserial glycosyltransferase *lgtC*. Mutation of this *Haemophilus lgtC*-homologue resulted in attenuated virulence in the infant rat model of invasive infection. The authors concluded that their studies indicated 'the rapidity, economy, and completeness with which whole genome sequences can be used to investigate the biology of pathogenic bacteria'. Akerley *et al.* [1] used a high-density transposon mutagenesis strategy to estimate that 38% of Rd genes are required for growth or viability in rich media. A number of putative essential genes (259) lacked defined functional roles. It was concluded that essentiality was not predicted by conservation across species, and that genes needed in one organism are not necessarily needed in another.

Given that the whole genome of *H. influenzae* was the first available, it is somewhat surprising that only two papers have been published to date reporting the use of the data to construct DNA arrays for transcriptome analysis. de Saizieu *et al.* [3] designed a high-density oligonucleotide probe array (Affymetrix chip) containing probes representing 106 *H. influenzae* genes and 100 *Streptococcus pneumoniae* genes, but only reported the results of probing with chemically biotinylated RNA from *S. pneumoniae* grown up in rich media. Not surprisingly, there was little cross-reactivity of the *S. pneumoniae* RNA with oligonucleotides representing *H. influenzae* genes. Gmuender *et al.* [5] used an Rd whole-genome Affymetrix chip to investigate the transcriptome of the bacterium exposed to DNA gyrase inhibitor antibiotics of two functional classes during growth in minimal media. Novobiocin inhibits the ATPase activity of the enzyme, indirectly changing the degree of DNA supercoiling, whilst ciprofloxacin obstructs supercoiling by inhibiting the DNA-cleavage-resealing reaction. With novobiocin the expression rate of many genes changed, reflecting that their transcription initiation is sensitive to DNA supercoiling. In contrast, ciprofloxacin mainly stimulated the expression of DNA repair systems in response to DNA damage. Time course and antibiotic concentrations were varied, and when all experiments were taken into account, 85% of transcribed genes were detected. A number of the genes whose expression levels changed were those of unknown function or conserved hypothetical genes. This study was extremely comprehensive in that samples were also analysed by two-dimensional electrophoresis (2DE). The intensity of signals on the Affymetrix chips was compared to that of the protein spots detected on 2DE gels and a correlation coefficient of 0.5 found. Possible reasons suggested for this low correlation were bias on cDNA synthesis, chip variation and the technical constraints of 2DE resulting in non-detection of proteins. However, this study amply demonstrates the power of *H. influenzae* gene arrays to give insight into the bacterial transcriptional consequences of antibiotic treatment.

Our laboratory has a long-standing interest in understanding both the basic biology and the

Copyright © 2002 John Wiley & Sons, Ltd. 

molecular basis of pathogenicity of *H. influenzae*. Advantages of working with *H. influenzae* as a model organism include the ease with which mutants can in general be made, the availability of an animal model that closely mimics human disease, and the availability of various *in vitro* surrogate models (tissue culture) of different phases of infection. To complement such techniques we have, in collaboration with Philip Butcher and Jason Hinds at the Bacterial Microarray Group at St. George's Hospital Medical School in London, constructed a whole-genome array of *H. influenzae* for transcriptome analysis. This follows on from our initial studies with a partial genome array of *H. influenzae* [2]. Primers were designed to amplify regions of all the ORFs — excluding the structural RNAs — chosen so that they would show minimum cross-hybridization within the *H. influenzae* genome. In addition to ORFs from *H. influenzae* Rd, we have included ORFs from other strains of *H. influenzae* (i.e. those not present in Rd) as well as ORFs from eukaryotic cells (as well as other bacterial species) to act as controls. We plan to use these arrays to identify genes that are expressed *in vivo*. It is firmly established that bacteria grown *in vitro* and *in vivo* express different sets of genes, reflecting adaptation to quite different environments, and in particular the need to survive the comparatively hostile environment provided by the host. The suggestion from the Tatusov analysis of the genome is that *H. influenzae* is adapted to growth in a more anaerobic environment. We shall determine the *in vivo* transcriptome of *H. influenzae* by isolating bacteria directly from experimentally infected animals and from *in vitro* surrogate models (tissue culture) and compare it to that relevant to bacteria grown *in vitro*. These experiments are not without practical and interpretative difficulties. First, there is the problem of isolating sufficient bacteria to obtain enough RNA for microarray analysis. This is dependent on the model system used. Second, the bacteria should ideally be free of host cell RNA; and third, methods requiring minimal sample manipulation will be essential to preserve material for analysis. The use of RNA-stabilizing agents, such as RNALater (Ambion), may be useful in this regard. In experiments of this kind, the conditions in which the comparator organisms are grown are of course crucial, and the choice of broth culture for 'baseline' is often rightly challenged. Considerable caution will be needed in the interpretation of results obtained from *in vivo* vs. broth-grown organisms, but it is reasonable to hope that data should be generated to identify genes or pathways to be given priority targeting in future studies. It should also be possible to formulate *in vitro* growth conditions that mimic the *in vivo* situation more closely, aiding for example identification of potential novel targets for antibiotic action. The availability of a whole-genome array for *H. influenzae* should also be invaluable in other areas of research: e.g. it will be possible to compare the transcriptome of bacteria grown under different *in vitro* growth conditions or in response to imposed environmental stresses. Alternatively, the response of wild-type and isogenic non-polar mutant strains under defined growth conditions can be compared. It should also be remembered that the transcriptome obtained is a snapshot. Comparatively few transcriptome studies have been formulated as time course analyses because of experimental or cost constraints. Such analysis may be crucial in establishing the significance of data. However, a systematic approach with good experimental design in combination with analysis of the metabolic pathways [8] should lead to the identification of the regulatory networks of *H. influenzae*. Additionally, transcriptome analysis should be combined with proteome analysis, as exemplified by Gmuender *et al.* [5], who used 2DE analysis.

Whilst at present there is only one publication describing the use of whole-genome arrays with *H. influenzae*, in the coming years we can predict that there will be increased interest once again in this organism, both for the reasons stated already, and because a non-typable strain is currently being sequenced (**http://www.microbial-pathogenesis.org/H.influenzae.86028/**). This should be available within 2 years, and it is expected that this will be used in the identification of potential vaccine as well as novel therapeutic targets. A DNA array of a non-typable strain will also be useful for genome-typing. In this technique, genomic DNA is hybridized to DNA arrays and the hybridization patterns used to identify genes present or absent in strains. This is useful in epidemiological investigations and for identifying potential pathogenicity islands. Non-typable strains are known to have a larger genome than Rd and be a relatively genetically diverse population, hence

Copyright © 2002 John Wiley & Sons, Ltd.

genome-typing may be a valuable tool for use with these bacteria.

### Acknowledgements

This work was supported by The Wellcome Trust (Grant No. 052172 to PRL). We also acknowledge BμG@S (the Bacterial Microarray Group at St George's) and The Wellcome Trust for funding their work.

### References

1. Akerley BJ, Rubin EJ, Novick VL, Amaya K, Judson N, Mekalanos JJ. 2002. A genome-scale analysis for identification of genes required for growth or survival of *Haemophilus influenzae*. *Proc Natl Acad Sci USA* **99**: 966–971.
2. Ali TR, Li M-S, Langford PR. 2002. Monitoring gene expression using DNA arrays. In *Haemophilus influenzae Protocols*, Mark A Herbert MA, Hood DW, Moxon ER (eds), Methods in Molecular Medicine, vol 71. Humana: Totowa, NJ (in press); Chapter 8.
3. de Saizieu A, Certa U, Warrington J, *et al.* 1998. Bacterial transcript imaging by hybridization of total RNA to oligonucleotide arrays. *Nature Biotechnol* **16**: 45–48.
4. Fleischmann RD, Adams MD, White O, *et al.* 1995. Whole-genome random sequencing and assembly of *Haemophilus influenzae* Rd. *Science* **269**: 496–512.
5. Gmuender H, Kuratli K, Di Padova K, Gray CP, Keck W, Evers S. 2001. Gene expression changes triggered by exposure of *Haemophilus influenzae* to novobiocin or ciprofloxacin: combined transcription and translation analysis. *Genome Res* **11**: 28–42.
6. Hood DW, Deadman ME, Allen T, *et al.* 1996. Use of the complete genome sequence information of *Haemophilus influenzae* strain Rd to investigate lipopolysaccharide biosynthesis. *Mol Microbiol* **22**: 951–965.
7. Hood DW, Deadman ME, Jennings MP, *et al.* 1996. DNA repeats identify novel virulence genes in *Haemophilus influenzae*. *Proc Natl Acad Sci USA* **93**: 11121–11125.
8. Karp PD, Ouzounis C, Paley S. 1996. HinCyc: A knowledge base of the complete genome and metabolic pathways of *Haemophilus influenzae*. *Intel Syst Mol Biol* **4**: 116–124 (**http://citeseer.nj.nec.com/karp00hincyc.html**)
9. Tatusov RL, Mushegian AR, Bork P, *et al.* 1996. Metabolism and evolution of *Haemophilus influenzae* deduced from a whole-genome comparison with *Escherichia coli*. *Curr Biol* **6**: 279–291.
10. Zwahlen A, Kroll JS, Rubin LG, Moxon ER. 1989. The molecular basis of pathogenicity in *Haemophilus influenzae*: comparative virulence of genetically-related capsular transformants and correlation with changes at the capsulation locus cap. *Microb Pathog* **7**: 225–235.

Copyright © 2002 John Wiley & Sons, Ltd. 
